# Improved γ-linolenic acid production in *Mucor circinelloides* by homologous overexpressing of delta-12 and delta-6 desaturases

**DOI:** 10.1186/s12934-017-0723-8

**Published:** 2017-06-21

**Authors:** Yao Zhang, Xiao Luan, Huaiyuan Zhang, Victoriano Garre, Yuanda Song, Colin Ratledge

**Affiliations:** 1 Colin Ratledge Center for Microbial Lipids, School of Agricultural Engineering and Food Science, Key Laboratory of Shandong Provincial Universities for Technologies in Functional Agricultural Products, Shandong University of Technology, 266 Xincun West Road, Zibo, Shandong 255000 People’s Republic of China; 2 School of Food Science and Technolgy, Jiangnan University, 1800 Lihu Avenue, Wuxi, Jiangsu 214122 People’s Republic of China; 3 Departamento de Genéticay Microbiologia (Unidad ASOCIADA al IQFR-CSIC), Facultad de Biologi, Universidad de Murcia, 30071 Murcia, Spain; 4 Department of Biological Sciences, University of Hull, Hull, UK

**Keywords:** Delta-6 desaturase, Delta-12 desaturase, GLA production, Homologous overexpression, *Mucor circinelloides*

## Abstract

**Background:**

γ-Linolenic acid (GLA) is important because of its nutritional value and medicinal applications. Although the biosynthetic pathways of some plant and microbial GLA have been deciphered, current understanding of the correlation between desaturases and GLA synthesis in oleaginous fungi is incomplete. In previous work, we found that a large amount of oleic acid (OA) had not been converted to linoleic acid (LA) or GLA in *Mucor circinelloides* CBS 277.49, which may be due to inadequate activities of the delta-12 or delta-6 desaturases, and thus leading to the accumulation of OA and LA. Thus, it is necessary to explore the main contributing factor during the process of GLA biosynthesis in *M. circinelloides*.

**Results:**

To enhance GLA production in *M. circinelloides*, homologous overexpression of delta-12 and two delta-6 desaturases (named delta-6-1 and delta-6-2, respectively) were analyzed. When delta-6 desaturase were overexpressed in *M. circinelloides*, up to 43% GLA was produced in the total fatty acids, and the yield of GLA reached 180 mg/l, which were, respectively, 38 and 33% higher than the control strain.

**Conclusion:**

These findings revealed that delta-6 desaturase (especially for delta-6-1 desaturase) plays an important role in GLA synthesis by *M. circinelloides*. The strain overexpressing delta-6-1 desaturase may have potential application in microbial GLA production.

**Electronic supplementary material:**

The online version of this article (doi:10.1186/s12934-017-0723-8) contains supplementary material, which is available to authorized users.

## Background

γ-Linolenic acid (GLA, 18:3, delta-6,9,12) is an important n-6 polyunsaturated fatty acid (PUFA) that has many health and medicinal effects for prevention and treatment of inflammatory disorders, diabetes, cardiovascular disorders, cancers, and some other diseases [1–4]. The traditional sources of GLA are from a relatively small number of plant seeds, including evening primrose (*Oenothera biennis* L.), borage (*Borago officinalis* L.) and blackcurrant (*Ribes nigrum* L.) [5–7] where the content of GLA may be up to 22% of the total fatty acids. However, GLA production from plant seeds is easily influenced by region, climate and seasons, resulting in variable quantities and qualities of the oils. Genetically-engineered safflower seeds are now available in which GLA may now be up to 70% of the total fatty acids [8, 9] but, because of the uncertainties surrounding the acceptability of genetically modified (GM) materials as neutraceuticals, there is continued interest in developing microbial oils over conventional sources. Thus, a promising alternative source comes from oleaginous microorganisms with high GLA contents [5, 10–12].

Generally in plant and microbial cells, the biosynthesis of PUFA derives from saturated stearic acid (18:0), which is first converted by delta-9 desaturase to yield oleic acid (OA, 18:1, delta-9). Then, delta-12 desaturase converts OA to linoleic acid (LA, 18:2 delta-9, 12). Subsequently, GLA is synthesized by introducing a double bond into LA using a delta-6 desaturase. Thus, three desaturases, the delta-9, delta-12 and delta-6, accomplish the desaturation processes in GLA synthesis [5, 13, 14]. Genes coding for those desaturases have been cloned from diverse organisms ranging from prokaryotes to higher eukaryotes, and overexpressed in several hosts, including microalgae, yeasts and plants [8, 13–16]. However, few researches on homologous expression of fungal desaturases have been reported.

*Mucor circinelloides*, a typical oleaginous filamentous fungus, has been widely used to investigate GLA production since the 1980s [12, 17]. Previously, we found that the proportion of OA, LA and GLA in total fatty acids of *M. circinelloides* were 38, 11, and 19%, respectively [18, 19]. This suggested that a large amount of OA had not been converted to LA or GLA, which may be due to inadequate activities of the delta-12 or delta-6 desaturases, and thus leading to the accumulation of OA and LA. Thus, it is necessary to explore the main contributing factor during the process of GLA biosynthesis in *M. circinelloides*. In this study, we report the cloning and homologous overexpression of the delta-12 and delta-6 desaturases derived from *M. circinelloides* itself with the aim of determining the correlation between fatty acid desaturases and GLA biosynthesis in oleaginous fungi.

## Results and discussion

### Construction of delta-12 and delta-6 desaturase genes overexpressing strains

**Fig. 1:**
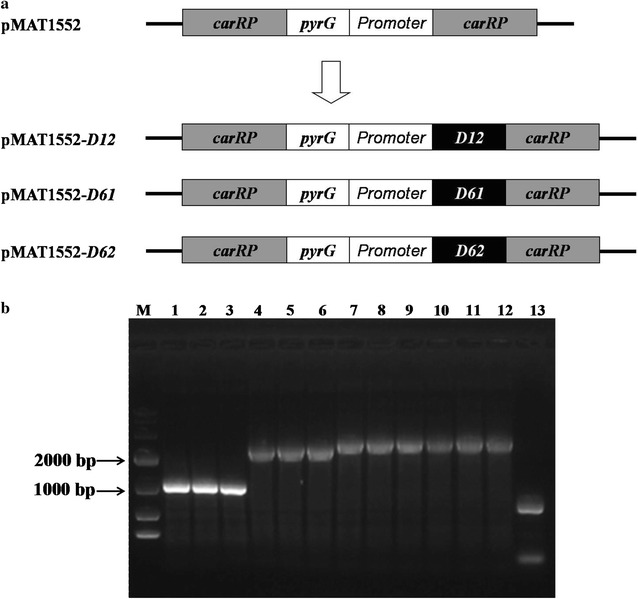
Overexpression of *D12*, *D61* and *D62* genes. **a** Structure of plasmids pMAT1552-*D12*, pMAT1552-*D61* and pMAT1552-*D62* for *D12*, *D61* and *D62* genes overexpressing in *M. circinelloides* are shown. *Black boxes* indicate coding region of the desaturase genes. **b** PCR amplification of genome of control strain (Mc-1552), wide-type strain (*M. circinelloides* CBS 277.49) and desaturase gene overexpressing strains (Mc-D12, Mc-D61 and Mc-D62) with the primers P3-F and P3-R. Every three transformants in the overexpressing strains and control strain were screened and cultivated in 500 ml flasks containing 100 ml K&R medium for 24 h at 30 °C with shaking at 150 rpm. Then, genomic DNA from these transformants was extracted and verified by PCR amplicfication. M, Gene Ruler DNA Ladder Mix, *1*–*3*, three transformants for Mc-1552, sequentially Mc-1552-1, Mc-1552-2, Mc-1552-3, *4*–*6*, three transformants for Mc-D12, sequentially Mc-D12-1, Mc-D12-2, Mc-D12-3, *7*–*9*, three transformants for Mc-D61, sequentially Mc-D61-1, Mc-D61-2, Mc-D61-3, *10*–*12*, three transformants for Mc-D62, sequentially Mc-D62-1, Mc-D62-2, Mc-D62-3, *13*, *M. circinelloides* CBS 277.49

Based on the genomic data of *M. circinelloides* CBS 277.49, we found one delta-12 desaturase gene, named *D12* (JGI accession number ID155268, 1351 bp), and two delta-6 (delta-6-1 and delta-6-2) desaturase genes, named *D61* (JGI accession number ID37214, 1738 bp) and *D62* (JGI accession number ID105367, 1575 bp). To determine if the delta-12 and delta-6 desaturases were involved in fatty acid accumulation, overexpressing strains of these genes were generated. The genes, *D12*, *D61* and *D62*, were cloned from the genome of *M. circinelloides* and subsequently inserted into the expression vector, pMAT1552, that contains the strong *zrt1* promoter, the *pyrG* gene as a selectable marker and flanking sequences corresponding to regions surrounding the carotenogenic *carRP* gene to allow its chromosomal integration by homologous recombination (Fig. 1a). The *D12*, *D61* and *D62* overexpressing plasmids, pMAT1552-*D12*, pMAT1552-*D61* and pMAT1552-*D62*, were transformed into the recipient strain MU402 and transformation and selection of the colonies were carried out as described by [20]. Three overexpressing and control transformants were selected at random and then confirmed by PCR analysis. Amplification was carried out using a primer pair (P3-F/R) that amplified the desaturase gene and 1000 bp sequences of the plasmid pMAT1552. Thus, the corresponding *D12* (2736 bp)*, D61* (2351 bp) and *D62* (2575 bp) gene fragments were amplified and, as expected, resulted in transformants genomes, whereas no desaturase gene fragment was amplified in the control strain (Mc-1552 carring the vector pMAT1552) and wild-type strain (*M. circinelloides* CBS 277.49) as shown in Fig. 1b. The PCR amplification results suggest that the *D12, D61* and *D62* genes had been respectively integrated into the genome of the fungus in the transformants.

**Fig. 2:**
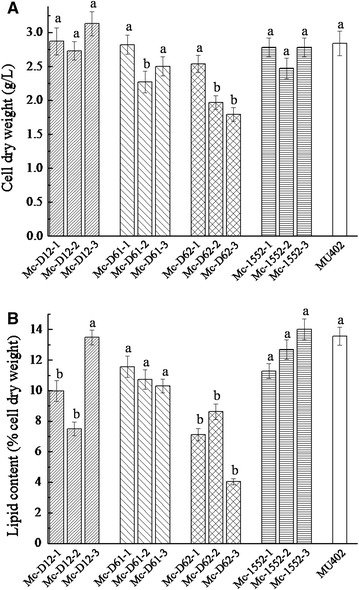
Cell growth and lipid content of different overexpressing transformants. Strains were cultured in 500 ml flasks containing 100 ml K&R medium for 72 h at 30 °C with shaking at 150 rpm. **A** Cell dry weight, **B** lipid content of cell dry weight. Values were mean of three independent fermentation experiments. *Error bars* represent the standard error of the mean

Overexpressing strains were grown in complete medium for 3–4 days in 1 l baffled flasks with 200 ml K&R medium. Compared with the control strain, Mc-1552, Mc-D12 and Mc-D61 had a similar biomass (Fig. 2A) and lipid content (Fig. 2B), suggesting that the knockout of *CarRP* gene and overexpression of delta-12 or delta-6-1 desaturase did not significantly affect the growth or lipid synthesis of the strains. However, Mc-D62 showed a large decrease in biomass and lipid content (Fig. 2). This indicated that the overexpression of delta-6-2 desaturase has an impact on growth and fatty acid synthesis. The possible explanation is that transformation procedure is stressful for the cells and this may cause mutations. In addition, based on the best growth and lipid production of different transformants, the strains (Mc-D12-3, Mc-D61-1, Mc-D62-2, Mc-1552-3) were selected for further analysis. The code number of the transformant is no longer marked below.

### Expression levels of delta-12 and delta-6 desaturase genes in the overexpressing strains

**Fig. 3:**
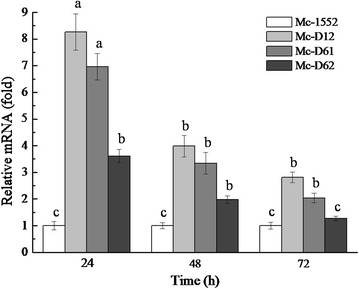
Determination of expression levels of *D12*, *D61* and *D62* genes by RT-qPCR in the overexpressing strains (Mc-D12, Mc-D61 and Mc-D62), and the control strain Mc-1552. Strains were grown in a 2 l fermenter with 1.5 l K&R medium, and the mycelium was harvested at 24, 48, and 72 h. Total RNA of strains at different time was extracted and verified by RT-qPCR. Values were mean of three independent fermentation experiments. *Error bars* represent the standard error of the mean

Real-time quantitative PCR were carried out to analyze the mRNA level of *D12*, *D61* and *D62* in the selected overexpression strains at 24, 48 and 72 h of growth in 2 l fermenter with K&R medium (Fig. 3). The mRNA expression level of *D12*, *D61* and *D62* was increased, respectively, by 8-, 7- and 3.6-fold at 24 h in overexpressing strains compared to the control, although it decreased with the incubation time. The fact that *D12*, *D61* and *D62* mRNA were maintained at elevated levels throughout the whole culture time confirmed that *D12*, *D61* and *D62* were overexpressed in the recombinant strains.

### Lipid accumulation in the delta-12 and delta-6 desaturase overexpressing strains

**Fig. 4:**
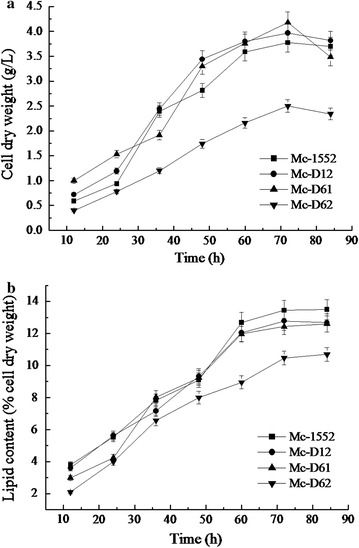
Cell growth and lipid content of *D12*, *D61* and *D62* overexpressing strains. Strains were cultured in a 2 l fermenter with the 1.5 l K&R medium during 84 h at 30 °C, pH 4.5, stirred at 500 rpm, with aeration at 0.5 vvm. In the fermentation process, biomass and lipid content were determined every 12 h. **a** Cell dry weight, **b** lipid content of cell dry weight. Values were mean of three independent fermentation experiments. *Error bars* represent the standard error of the mean

Growth and lipid accumulation in *D12*, *D61* and *D62* overexpressing strains were compared to the control strain over 72 h growth in a 2 l fermenter. Overall, the growth patterns of different strains were similar (Fig. 4a): the mycelia grew rapidly during the first 48 h after which the growth entered into stationary phase, and then after 72 h, growth ceased. Except for Mc-D62, other overexpressing strains, Mc-D12 and Mc-D61, had a similar amount of biomass compared with the control strain, Mc-1552. Manipulation of the expression of delta-12 and delta-6 desaturase genes had no significantly effect on the total lipid content (Fig. 4b), whereas it had an obvious influence on the fatty acid composition (Table 1).

**Table 1:** The fatty acid composition in DELTA-12 and DELTA-6 desaturase genes overexpressing strains

Strains	Time (h)	Fatty acid composition (relative %, w/w)^a^				
		16:0	18:0	18:1	18:2 (LA)	18:3 (GLA)
Mc-1552						
	24	23.1 ± 0.5	4.0 ± 2.0	23.2 ± 8.7	17.4 ± 5.5	30.0 ± 7.1
	36	23.8 ± 0.5	3.6 ± 0.1	26.2 ± 0.1	14.8 ± 1.6	31.0 ± 1.2
	48	21.1 ± 0.2	2.7 ± 1.3	28.3 ± 2.6	14.5 ± 1.3	31.1 ± 2.6
	60	18.2 ± 0.9	2.2 ± 0.6	24.6 ± 0.2	14.3 ± 1.8	31.2 ± 0.1
	72	17.1 ± 0.6	2.0 ± 0.5	24.0 ± 3.6	14.3 ± 3.2	31.2 ± 0.4
Mc-D12						
	24	23.0 ± 8.4	3.0 ± 0.9	12.1 ± 0.8	24.0 ± 1.4	28.2 ± 1.7
	36	21.5 ± 3.1	2.7 ± 0.1	20.6 ± 3.0	21.5 ± 1.8	28.1 ± 2.3
	48	20.4 ± 1.0	2.2 ± 0.3	22.0 ± 2.0	20.8 ± 1.2	28.6 ± 2.7
	60	19.1 ± 0.1	1.8 ± 0.1	22.4 ± 0.3	20.6 ± 0.1	28.6 ± 0.4
	72	17.7 ± 0.2	1.6 ± 0.3	21.8 ± 1.3	20.1 ± 2.1	29.0 ± 0.7
Mc-D61						
	24	22.1 ± 0.2	ND	16.5 ± 0.1	17.5 ± 0.1	41.9 ± 0.4
	36	18.0 ± 0.1	ND	22.2 ± 0.1	12.3 ± 0.0	42.2 ± 0.1
	48	16.8 ± 0.2	1.4 ± 0.0	22.1 ± 0.2	13.3 ± 0.1	42.6 ± 0.0
	60	16.2 ± 0.1	1.3 ± 0.3	22.1 ± 0.2	14.4 ± 0.2	42.8 ± 0.2
	72	14.9 ± 0.1	ND	22.1 ± 0.2	14.2 ± 0.1	43.0 ± 0.6
Mc-D62						
	24	22.5 ± 0.1	4.2 ± 0.0	19.3 ± 0.1	16.6 ± 0.1	36.6 ± 0.2
	36	22.0 ± 0.5	3.6 ± 0.3	21.5 ± 0.2	15.4 ± 0.1	36.5 ± 0.1
	48	21.3 ± 0.1	3.2 ± 0.1	22.1 ± 0.2	14.4 ± 0.1	37.0 ± 0.1
	60	20.2 ± 0.2	3.0 ± 0.1	22.4 ± 0.0	13.6 ± 0.0	37.0 ± 0.1
	72	18.0 ± 0.1	2.7 ± 0.3	22.8 ± 0.2	13.3 ± 0.1	37.1 ± 0.6

*ND* not detected

^a^Strains were cultured in a 2 l fermenter with the 1.5 l Kendrick medium during 72 h. The fatty acid composition were displayed at different point times. The values are means ± standard deviations of two independent experiments

When compared with the control strain, Mc-1552, OA (18:1) decreased by 10% while LA (18:2) increased by 41% in Mc-D12 (Table 1), suggesting that the overexpression of delta-12 desaturase leads to the conversion of more OA to LA which should then become the substrate for GLA synthesis. However, the content of GLA in Mc-D12 decreased rather than increasing compared to the control, which suggests that delta-12 desaturase is not the rate-limiting step in GLA biosynthesis. Kelder et al. [21] has found that the n-6 series of fatty acids in rat L cells showed a downward trend upon overexpression of *Mortierella alpina* delta-12 desaturase which is similar to what was seen in this study. The reason for this result was likely that the increase in the membrane fluidity with the increase of LA content by delta-12 desaturase expression might cause the suppression of delta-6 desaturase.

As opposed to the overexpression of delta-12 desaturase, compared to the control strain Mc-1552, the GLA content of Mc-D61 and Mc-D62 were, respectively, increased by 38 and 19% whilst the other fatty acids essentially remained unchanged thereby indicating that delta-6 desaturase probably plays an important role in GLA synthesis. However, the increment of GLA increase for Mc-D62 was about half of that for Mc-D61, and with a lower biomass and total lipid content. This indicated that the delta-6-2 desaturase may play a supplementing role for delta-6-1 desaturase in the synthesis of GLA. In addition, the *D61* overexpressing strain, Mc-D61, finally reached 180 mg GLA/l, which was 33% greater than that of the control strain Mc-1552. Notably, the proportion of GLA in total lipid content for Mc-D61 reached 43%, was much higher than that (18–19%) in the strain of *M. circinelloides* used for the commercial production of GLA in the 1980s [12]. This indicates that the strain Mc-D61 may have great industrial prospects in future if the cell total lipid content can be improved.

Surprisingly in our study, the effects of these two delta-6 fatty acid desaturases were quite different. The two delta-6 desaturases shared only 22.8% homology and their phylogenetic positions were far apart from each other, which is in accordance with the delta-6 genes in *M. circinelloides* HUT1121 [22]. Similarly, a low degree of homology particularly for fungal delta-6 desaturases is evident when comparing the genes from *Mortierella* [23] and *Mucor* [24]. Although the sequence of delta-6-2 desaturase from *M. circinelloides* was close to the delta-6 desaturase of the high GLA-producing borage (*B. officinalis*), it is not efficient in GLA synthesis in *M. circinelloides*. The physiological functions of delta-6-2 desaturase need further investigation.

### Co-expression of delta-12 and delta-6 desaturase genes

To further explore the combined effect of delta-12 and delta-6 desaturases on GLA production, co-expression of *D12* and *D61* were constructed in *M. circinelloides*. The *D12* gene was inserted into the expression vector pLEU4 containing *Leu*A gene and then transformed into the recipient strain MU402 with the plasmid, pMAT-D61, overexpressing *D61* gene. Finally, a recombinant strain, Mc-D1261, co-expressing *D12* and *D61* was obtained. The mRNA expression levels of *D12* and *D61* were significantly increased by 8- and 6.5-fold in the co-expressing strain Mc-D1261 compared to the control stain Mc-1552, which was similar with the result of its single overexpression.

**Fig. 5:**
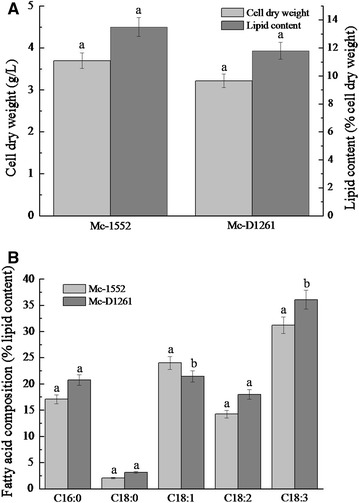
Cell growth and lipid accumulation in *D12* and *D61* co-expressing strain (Mc-D1261) and the control strain Mc-1552. Strains were cultured in a 2 l fermenter with the 1.5 l K&R medium for 72 h at 30 °C, pH 4.5, stirred at 500 rpm, with aeration at 0.5 vvm. **A** Cell dry weight and lipid content of cell dry weight, **B** the fatty acid composition. Values were mean of three independent fermentation experiments. *Error bars* represent the standard error of the mean

Growth and lipid accumulation in the co-expression strain, Mc-D1261, was examined in a 2 l fermenter using K&R medium at 72 h (Fig. 5A). Mc-D1261 had a slightly decrease in biomass compared with the control strain Mc-1552 but no significant change in its total lipid content. Compared with the control strain Mc-1552, the OA content of Mc-D1261 was decreased by 12%, whereas the LA and GLA content of Mc-D1261 were increased by 26.5 and 15.5%, respectively. Unexpectedly, the increase of GLA by co-expression of *D12* and *D61* was lower than that achieved by overexpression of the single *D61*. The reason for this might be that delta-12 desaturase is not rate-limiting in LA production and also, as same as the overexpression of single *D12*, the expression of delta-12 desaturase may have restrained the expression of delta-6 desaturase due to the changes of membrane fluidity. Further study for the introduction of a mutation that cancels the repression of delta-12 desaturase would be needed to solve this problem.

## Conclusion

Delta-12 and delta-6 fatty acid desaturases were overexpressed by homologous recombination in the oleaginous fungus *M. circinelloides* CBS 277.49 to enhance the production of GLA. Delta-6 desaturase overexpressing *M. circinelloides* generated in this study had up to 43% GLA in the final lipid with a final yield of 180 mg GLA/l. These values are, respectively, 38 and 33% higher than those in the control strain. Thus, we can conclude that delta-6 desaturase (especially for delta-6-1 desaturase) plays an important role in GLA synthesis by *M. circinelloides*. The *D61* overexpressing strain, Mc-D61, may have potential application in microbial GLA production.

## Methods

### Strains, plasmids and culture conditions

*Mucor circinelloides* CBS 277.49 was used as the source of delta-12 desaturase gene, *D12*, and two delta-6 desaturase genes, *D61* and *D62*. The leucine and uracil double auxotroph strain, MU402 [25], was used as the recipient strain in transformation experiments to overexpress the desaturase genes. *Escherichia coli* strain Top 10 was used for all cloning experiments and grown in lysogeny broth at 37 °C. Plasmids pMAT1552 and pLEU4 [26] were used as the cloning and expression vectors. Cultures were grown at 26 °C in YPG [27]. Media were supplemented with leucine (20 μg/ml) or uridine (200 μg/ml) when required. The pH was adjusted to 4.5 and 3 for mycelia and colonial growth, respectively.

The recombinant strains Mc-D12 (*D12* overexpresssion strain), Mc-D61 (*D61* overexpression strain), Mc-D62 (*D62* overexpression strain), Mc-D1261 (*D12* and *D61* coexpresssion strain), and Mc-1552 (strain carrying the vector pMAT1552) as the control were initially cultivated in 500 ml flasks containing 100 ml K&R medium [28] for 24 h at 30 °C with shaking at 150 rpm and then inoculated at 10% (v/v) into a 2 l fermenter (BioFlo/CelliGen115, New Brunswick Scientific, Edison, NJ, USA) containing 1.5 l K&R medium modified to contain 80 g glucose/l. Fermenters were held at 30 °C, stirred at 500 rpm, with aeration at 0.5 vvm. The pH was maintained at 4.5 by automatic addition of 1 M NaOH or 1 M HCl, and medium was supplied with leucine (20 μg/ml) or uracil (200 μg/ml) when required.

### Plasmids construction

The vector, pMAT1552, which was used for construction of *D12*, *D61* and *D62* overexpression plasmids, contains only a *Xho*I restriction site between its promoter and homology arm *CarRP* sequence. To facilitate subsequent cloning screening, a *CarRP* sequence was amplified by using primers Car-F/R (Additional file 1: Table S1), with another *Xba*I restriction site inserted between the *Xho*I and SacII sites of original *CarRP* fragment. The amplification product was isolated and ligated into the vector pMD19T-simple to generate an intermediate vector pMD19T-*CarRP*.

The genes, *D12*, *D61* and *D62*, were isolated by PCR amplification from the genome of *M. circinelloides* CBS 277.49 with separate primers D12-F/R, D61-F/R and D62-F/R (Additional file 1: Table S1) that introduced the *Xho*I and *Xba*I restriction sites, respectively, at the 5′- and 3′-ends of desaturase genes. The products were digested by *Xho*I and *Xba*I and ligated into the similarly digested intermediate vector pMD19T-*CarRP*. The ligation mixture was used to transform chemically competent *E. coli* Top 10 cells. The plasmids isolated from these transformants were verified by restriction analysis and the gene sequences were confirmed by DNA sequencing. The plasmids with the correct sequences of *D12*, *D61* and *D62* were named pMD19T-*CarRP*-D12, pMD19T-*CarRP*-D61 and pMD19T-*CarRP*-D62, respectively. Because *D12* and *D61* genes contain one internal *Xho*I site, site-directed mutagenesis was performed to eliminate these sites while keeping the encoded amino acid unchanged. The resulting plasmids were digested with *Xho*I and *Eco*RI and ligated into the similarly digested expression vector pMAT1552. The ligation mixture was used to transform chemically competent *E. coli* Top 10 cells. The plasmids isolated from these transformants were verified by restriction analysis and the gene sequences were confirmed again by DNA sequencing. The plasmids with the correct sequences of *D12*, *D61* and *D62* were named pMAT-D12, pMAT-D61 and pMAT-D62, respectively. Transformation was carried out by electroporation-mediated procedure as described previously [29].

To achieve the co-expression of *D12* and *D61*, another *D12* gene expression plasmid was constructed using the vector pLEU4. The *D12* gene was isolated by PCR amplification from the plasmid pMAT-D12 and then cloned into the expression vector pLEU4 with primers D12′-F/R (Additional file 1: Table S1). The amplification product was digested by *Kas*I and *Sal*I and ligated into the similarly digested expression vector pLEU4. The ligation mixture was used to transform chemically competent *E. coli* Top 10 cells. The plasmids isolated from these transformants were verified by restriction analysis and the gene sequences were confirmed by DNA sequencing. The plasmids with the correct sequences of *D12* was named pLEU-D12. Then, the plasmids pLEU-D12 and pMAT-D61 were simultaneously transformed into competent MU402 cells.

### Gene expression and RT-qPCR analysis

*D12*, *D61* and *D62* overexpressing transformants were cultivated in YNB for 3 days before extracting the genomic DNA. The mycelia was harvested by suction filtration and washed twice with sterile water. The genomic DNA was extracted by following the protocols of the DNA Quick Plant System (Tiangen). PCR amplication was used to verify whether *D12*, *D61* or *D62* had integrated into the genome of *M. circinelloides* with the universal primers P3-F/R (Additional file 1: Table S1).

For reverse transcription-quantitative PCR (RT-qPCR) analysis, strains were grown in a 2 l fermenter with K&R medium, and the mycelium was harvested at 24, 48, and 72 h. Total RNA of *M. circinelloides* was extracted with Trizol after grinding under liquid N_2_ and reverse-transcribed using the Prime ScriptRT reagent kit (Takara) according to the manufacturer’s instructions. RT-qPCR was performed using primers (Additional file 1: Table S1) on CFX Connect Real-Time System (Bio-Rad) with iTaq Universal SYBR Green PCR Supermix (Bio-Rad) according to the manufacturer’s instruction. The mRNA expression level was normalized to levels of 18S rRNA mRNA, and the results were expressed as relative expression levels. The data were quantified by the method of 2^−ΔΔCt^.

### Determination of cell dry weight and fatty acid analysis

Biomass was harvested on a weighed filter paper by filtration through a Buchner funnel under reduced pressure and washed three times with distilled water, frozen overnight at −80 °C and then freeze-dried. The weight of the biomass was determined gravimetrically.

Biomass was collected by filtration and dried by lyophilizer. 20 mg dry weight was taken for cell fatty acids extraction. Pentadecanoic acid (15:0) was added into the freeze-dried cells as an internal standard. The total fatty acids were extracted with chloroform/methanol (2:1, v/v) and methylated with 10% (v/v) methanolic HCl at 60 °C for 3 h. The resultant fatty acid methyl esters were extracted with n-hexane and were analyzed by GC equipped a 30 m × 0.32 mm DB-Waxetr column with 0.25 µm film thickness. The program was as follows: 120 °C for 3 min, ramp to 200 °C at 5 °C per min, ramp to 220 °C at 4 °C per min, hold 2 min.

### Statistical analysis

A statistical analysis was carried out using SPSS 16.0 for Windows (SPSS Inc. Chicago, IL). The mean values and the standard error of the mean were calculated from the data obtained from three independent experiments. The differences between the means of the test were evaluated by Student’s *t* test, and *P* < 0.05 was considered as significantly different.

## Additional file

**Additional file 1: Table S1.** Primer sequences used in this study.

## Abbreviations

GLA: γ-linolenic acid
PUFA: polyunsaturated fatty acid
GM: genetically modified
OA: oleic acid
LA: linoleic acid
GC: gas chromatography
