# Supplementary material for: Improved γ-linolenic acid production in Mucor circinelloides by homologous overexpressing of delta-12 and delta-6 desaturases
Source: Microb Cell Fact. 2017 Jun 21;16:113. doi: 10.1186/s12934-017-0723-8 (PMC5480167; doi:10.1186/s12934-017-0723-8)
Supplement: Supplementary file 1 — Additional file 1: Table S1. Primer sequences used in this study. [file 12934_2017_723_MOESM1_ESM.docx]

Table S1 Primers used in this study

| Primer | Sequence (5’-3’)^a^ | Restriction enzyme | Application |
| --- | --- | --- | --- |
| Car-F | CTCGAGCCGCGGCAATCTAGATTGCGACTAG | *Xba*I |  |
| Car-R | CATGATTACGAATTCGAGCTCGGTACCC |  |  |
| D12-F | CCGCCTCGAGATGGCAACCAAGAGAAACG | *Xho*I |  |
| D12-R | GCGGTCTAGATTAGTTCTTGAAGAAGACG | *Xba*I |  |
| D61-F | CCGCCTCGAGATGAGCAGCGACGTAGGAGC | *Xho*I |  |
| D61-R | GCGGTCTAGATTAGAGCATCTTTTTGATGA | *Xba*I |  |
| D62-F | CCGCCTCGAGATGCCCCCAAACACTG | *Xho*I |  |
| D62-R | GCGGTCTAGACTAATGAGCATGTTCCTTCTC | *Xba*I |  |
| MutD12-F | GGATTGTTCCCGAGTGATACTAAAC |  | Site-directed mutagenesis |
| MutD12-R | TTTAGTATCACTCGGGAACAATCC |  |  |
| MutD61-F | ACACACGAGCTGAATTGGC |  | Site-directed mutagenesis |
| MutD61-R | GCCAATTCAGCTCGTGTGT |  |  |
| D12’-F | AAGGGGCGCCATCATCATCGATGTTTGTGCTGTCA | *Kas*I |  |
| D12’-R | ACGCGTCGACACCCGGGCATGTGTAACAGTGCATT | *Sal*I |  |
| P3-F | AGAGCACTTTTTAGCGCCTCTGGAT |  |  |
| P3-R | TCATTTTTCCCTGTCTGCAATGTGT |  |  |
| D12q-F | GCCCACATCAAGAAGGCTCT |  | RT-PCR analysis |
| D12q-R | CTTCGACAAAGCGACAGCTC |  |  |
| D61q-F | GGCTAATTGCTACGTTGGCG |  | RT-PCR analysis |
| D61q-R | TACACACAAGCAGGGTGGTC |  |  |
| D62q-F | ATGTGGATTGCCCAGAGTGG |  | RT-PCR analysis |
| D62q-R | TTGACCAAAGGCACGCATTG |  |  |

^a^ Restriction enzyme sites are underlined.
